# IL-10 transcription is negatively regulated by BAF180, a component of the SWI/SNF chromatin remodeling enzyme

**DOI:** 10.1186/1471-2172-13-9

**Published:** 2012-02-15

**Authors:** Andrea L Wurster, Patricia Precht, Kevin G Becker, William H Wood, Yongqing Zhang, Zhong Wang, Michael J Pazin

**Affiliations:** 1 Laboratory of Molecular Biology and Immunology, National Institute on Aging Intramural Research Program, National Institutes of Health, Baltimore, USA; 2 Gene Expression and Genomics Unit, National Institute on Aging Intramural Research Program National Institutes of Health, Baltimore, USA; 3 Cardiovascular Research Center, Massachusetts General Hospital, Harvard Medical School, Boston, MA, USA; 4 Current address: National Human Genome Research Institute, NIH, 5635 Fishers Lane, Bethesda, MD 20892, USA; 5 Current address: National Institute of Allergy and Infectious Diseases NIH, 6700B Rockledge Drive, Bethesda, MD 20892-7616, USA

## Abstract

**Background:**

SWI/SNF chromatin remodeling enzymes play a critical role in the development of T helper lymphocytes, including Th2 cells, and directly program chromatin structure at Th2 cytokine genes. Different versions of SWI/SNF complexes, including BAF and PBAF, have been described based on unique subunit composition. However, the relative role of BAF and PBAF in Th cell function and cytokine expression has not been reported.

**Results:**

Here we examine the role of the PBAF SWI/SNF complex in Th cell development and gene expression using mice deficient for a PBAF-specific component, BAF180. We find that T cell development in the thymus and lymphoid periphery is largely normal when the BAF180 gene is deleted late in thymic development. However, BAF180-deficient Th2 cells express high levels of the immunoregulatory cytokine IL-10. BAF180 binds directly to regulatory elements in the Il-10 locus but is replaced by BAF250 BAF complexes in the absence of BAF180, resulting in increased histone acetylation and CBP recruitment to the IL-10 locus.

**Conclusions:**

These results demonstrate that BAF180 is a repressor of IL-10 transcription in Th2 cells and suggest that the differential recruitment of different SWI/SNF subtypes can have direct consequences on chromatin structure and gene transcription.

## Background

In T cells, chromatin structure can be dependent on cell fate, cell activation, or both. This is well illustrated in the case of the Th2 cytokine cluster, containing the Th2 cytokines IL-4, IL-5 and IL-13 [1,2]. The Th2 cytokines are exclusively expressed in Th cells that have differentiated into the Th2 lineage and only upon T cell activation. DNase I hypersensitivity site (DHS) mapping of the cytokine loci from different Th subsets revealed dramatic changes in chromatin accessibility across the locus in Th2 cells compared to other Th lineages and undifferentiated Th precursors (Thps); typically, DHS are nucleosome-free regions created by chromatin remodeling proteins directed by the binding of transcription factors [2,3]. Many of the DHS were subsequently determined both genetically and biochemically to be enhancer and silencer elements important to Th2 cytokine expression and were marked with lineage-specific changes in histone modifications [2,3]. Although changes in nuclease accessibility across cytokine loci in response to differentiation and activation signals have been well documented, less is known about to the specific enzymes responsible for these changes [4].

IL-10 was originally described as a Th2-specific cytokine, and the IL-10 gene is located on a different chromosome from the Th2 cytokine gene cluster [5]. Like the Th2 cytokines, IL-10 expression in Th2 cells is accompanied by changes in the accessibility in the IL-10 locus directed by both lineage and activation-specific signals [6-8]. More recently the expression of IL-10 has been shown to be less restricted and more plastic than the classical Th2 cytokines. Both Th1 and Th17 cells can express IL-10 under specific conditions, while the newly described Th9 subset produces high levels of IL-10 along with IL-9 [9-11]. Biologically, IL-10 exhibits strong immunosuppressive effects and serves to attenuate immune responses. This is illustrated in the development of profound inflammatory bowel disease and exaggerated immune responses in IL-10-deficient mice [12]. Indeed, some Treg cell populations, critical for the negative regulation of immune responses, mediate their activity through IL-10 expression [13,14]. A number of studies have linked genetic variants at the IL-10 gene to human disease [15-18].

ATP-dependent remodeling enzymes contain SWI2/SNF2-like ATPase subunits, and these ATPases couple the hydrolysis of ATP to changes in chromatin structure. SWI/SNF, Mi2, ISWI, and other ATP-dependent remodeling enzymes are classified into subfamilies based upon homology of the ATPase subunit [4,19,20]. These remodeling enzymes appear to both activate and repress gene expression [4,21-25]. SWI/SNF complexes are arguably the best-characterized ATP-dependent remodeling enzymes in T lymphocytes, with demonstrated functions in both early T cell development and T cell effector function [4,26]. Mammalian SWI/SNF complexes contain one copy of either the BRG1 or Brm ATPase, and approximately 10 additional accessory subunits to form complexes that are generally over a megadalton in size. Two versions of SWI/SNF complex, BAF and PBAF, have been described, based on subunit composition [25,27-32], as well as other complexes specific to ES cells and neurons [33,34]. For example, BAF complexes contain either the BRG1 or Brm ATPase, and either BAF250a or BAF250b. PBAF complexes contain BAF180, BAF200 and the BRG1 ATPase but not Brm. Importantly, BAF and PBAF complexes appear to regulate different target genes [29,31].

Previous we identified BAF250-containing BAF complexes as important chromatin remodelers of cytokine loci in T cells [24,35] and, in ES cells, regulators of pluripotency and self-renewal [36,37]. BAF complexes have established roles in cell cycle [38] and tumorigenesis [39]. PBAF complexes are known to be important in coronary development [40,41]. However, a role for PBAF complexes in T cell differentiation and effector function has not been explored. The PBAF specific SWI/SNF component, BAF180 or polybromo (Pbrm1), appears to direct the regulation of a unique set of target genes [42]. BAF180-deficient mice have defects in cardiac development that include the specific misregulation of retinoic acid-induced genes [41]. BAF180 also plays an important role in the regulation of the cell cycle due, at least in part, to its ability to activate the transcription of the cell cycle regulator p21 [43,44]. BAF180 mutations have been identified in breast and renal cancers suggesting BAF180 is a tumor suppressor gene [44,45]. Mutation of another PBAF component, BAF200/Arid2, is found in hepatocellular carcinoma [46]. The BAF180 protein includes an HMG DNA binding domain, two bromo-adjacent homology domains involved in protein-protein interactions and an array of six tandem bromodomains shown to bind to specific acetylated histone residues [42]. Recruitment of BAF180 to its specific gene targets has been suggested to involve interactions with other proteins, including transcription factors, and the recognition of specific histone signatures.

In this study we examine the role of BAF180-containing PBAF complexes in CD4+ T cells. Using cells from mice conditionally deleted for BAF180 in T cells, we found that overall thymus and peripheral T cell development was intact. Additionally, the ex-vivo differentiation of CD4 T helper cells into different effector fates was not absolutely dependent on BAF180. However, BAF180 appeared to function as a repressor of the immunoregulatory cytokine, IL-10, in Th2 cells. BAF180 bound directly to regulatory elements in the Il-10 locus but was replaced by BAF250-containing BAF complexes in the absence of BAF180, resulting in increased histone acetylation and CBP recruitment to the IL-10 locus. These results suggest that the differential recruitment of different SWI/SNF subtypes (BAF and PBAF) can have direct consequences on gene transcription and cell fate in T cells.

## Methods

### Mice

The generation of BAF180 conditional KO ES cell lines and mice are similar to that applied in BAF250a KOs [36]. The BAF180 genomic sequences used for generating the initial KOs [40] were subcloned into the conditional KO vector [36]. The generation of BAF180 conditional KO ES cell lines and mice are similar to that applied in BAF250a KOs [35]. Briefly, two FRT and two loxp sites, together with a polylinker sequence, were engineered into a vector containing a promoterless β-geo trapping cassette derived from pGT1. DNA fragments ~4 kb in length were PCR-amplified from genomic DNA 5' and 3' of exon 11 of BAF180 and inserted into the targeting vector as applied previously for conventional BAF180 KO. A 0.5-kb fragment containing exon 11 was PCR-amplified and inserted upstream of the β-geo trapping cassette. The BAF180 conditional knockout vector was linearized by NotI digestion and electroporated into E14 feeder-independent ES cells to generate heterozygous ES lines after selection in G418. Targeted ES lines were confirmed by Southern analysis. Spe I digestion produces a 7.8-kb fragment for wild type (WT) allele and a 5.9-kb fragment for a mutant allele. The probe used is located between the Spe I sites and distal to the 5' recombination region as applied previously for conventional BAF180 KO. BAF180 heterozygous mice were obtained as described and these mice were kept in a B6-129 mixed genetic background before they were crossed to CD4-Cre mice. All other procedures were as described previously [40]. CD4-Cre mice were obtained from Taconic. Animal approval was from the NIA ACUC, protocol ASP-365-MJP-Mi, and all experiments conform to the relevant regulatory standards.

### Cell culture

Mouse T cells were isolated and cultured essentially as described previously [24,47]. Naïve Thp cells were purified from lymph node and spleens by using CD4 + CD62+ T cell isolation kit (Miltenyi) to 95% purity. Lymphocytes were cultured in RPMI 1640 supplemented with 10% FCS, 100 U/ml penicillin, 100 μg/ml streptomycin, 1 mM Sodium Pyruvate, 2 mM L-glutamine, 25 mM Hepes, 50 μM β-mercaptoethanol. Purified naïve Thp cells were plated onto anti-CD3 (1 μg/ml), anti-CD28 (2 μg/ml) coated plates at 1-2 × 10^6 ^in the presence of 10 ng/ml IL-4, 10 μg/ml anti-IFNγ (Th2 conditions) or 1 ng/ml IL-12, 10 μg/ml anti-IL-4 (Th1 conditions). IL-2 (100 U/ml) was added 24 h later. Cultures were expanded in IL-2 (100 U/ml) 3 days after initial culture. For Th17 differentiation naïve Thp cells were cultured with soluble anti-CD3 (4 μg/ml), soluble anti-CD28 (1 μg/ml), 10 μg/ml of both anti-IL-4 and anti-IFNγ, 100 ng/ml IL-6, 10 ng/ml IL-1β and 1 ng/ml TGF-β. Th17 cells were expanded in 10 ng/ml IL-23. Proper differentiation was confirmed by intracellular cytokine staining for Th lineage signature cytokines and mRNA analysis.

### FACS analysis

Cells were stained, then analyzed on a FACSCalibur (BD Biosciences) using CellQuest software and standard methods. Fluorescently labeled antibodies to CD4, CD62L, CD44, CD8, CD3, and B220 were all purchased from BD Pharmingen. Before staining, Fc receptors were blocked with anti-CD16/32 Ab (BD Pharmingen). Negative controls consisted of isotype-matched, conjugated, nonspecific Abs (BD Pharmingen). Intracellular cytokine staining was performed using the Intracellular Cytokine Staining Kit (BD Pharmingen). Briefly, the cells were stimulated with PMA and Ionomycin for 4 hours in the presence of brefeldin A. The cells were fixed with paraformaldehyde, permeabilized and subsequently stained for cytokine expression using antibodies purchased from BD Pharmingen.

### Cell proliferation

Naïve Thp cells were plated in triplicate at 10,000 cells per well of a 96 well plate in the presence of the indicated amounts of plate bound anti-CD3 and 1 μg/ml anti-CD28 (Pharmingen). Proliferation was assessed after 72 h incubation using CyQuant

Cell Proliferation Assay (Invitrogen). Fluorescence was measured on a CytoFluor 4000 fluorescent plate reader. Each bar is the average and standard deviation of three wells and representative of three independent experiments.

### RNA analysis

Total RNA was purified using RNeasy columns (Qiagen). cDNA was made using iScript (BioRad) according to the manufacturer's instructions. Steady state mRNA levels of indicated genes were determined by real time PCR using SYBR green (Qiagen) on an ABI 7500. Ongoing transcription of IL-10 was measured by detection of the IL-10 primary (unspliced) transcript. Expression levels were normalized to mTBP [24,48] or m-actin as indicated. Oligo sequences are in Table 1; IL-10 primary transcript oligo pair is IL-10 pri.

**Table 1:** Primers for steady-state mRNA and primary transcript

Locus	Primer name	Primer 1	Primer 2
IL-4	MP 588	ACAGGAGAAGGGACGCCAT	GAAGCCCTACAGACGAGCTCA

IL-13	MP 590	AGACCAGACTCCCCTGTGCA	TGGGTCCTGTAGATGGCATTG

IFN-γ	MP 592	GGATGCATTCATGAGTATTGC	CCTTTTCCGCTTCCTGAGG

IL-5	MP 779	AGCACAGTGGTGAAAGAGACCTT	TCCAATGCATAGCTGGTGATTT

IL-17A	MP 1018	ATCAGGACGCGCAAACATG	GCAGCAACAGCATCAGAGACA

IL-17 F	MP 1020	ATTCCAGAACCGCTCCAGTTC	GGGTCTCGAGTGATGTTGTAATCC

IL-10	MP 1016	GGCGCTGTCATCGATTTCTC	GCTCCACTGCCTTGCTCTTATTT

IL-10 pri	MP 1082	CCAATGGGTACTAACCAGATGCT	AATTCATTCATGGCCTTGTAGACA

T-bet	MP 602	CAACAACCCCTTTGCCAAAG	TCCCCCAAGCAGTTGACAGT

GATA3	MP 604	AGAACCGGCCCCTTATCAA	AGTTCGCGCAGGATGTCC

RORγT	MP 1564	CTGTTTCGAGCCTTGGGCT	AAAGTCAAATATGGAGCTGATGAGC

actin	MP 598	AGAGGGAAATCGTGCGTGAC	CAATAGTGATGACCTGGCCGT

TBP	MP 935	CTTCGTGCAAGAAATGCTGAATAT	TGTCCGTGGCTCTCTTATTCTCA

### Illumina oligonucleotide microarray analysis

Three pairs of RNA's from resting and stimulated WT and BAF180-/- Th2 cells were analyzed by microarray analysis. Transcriptional profiling was determined using Illumina beadchips as described previously [49]. Briefly, total RNA was used to generate biotin-labeled cRNA using the Illumina TotalPrep RNA Amplification Kit. A total of 0.75 ug of biotin-labeled cRNA was hybridized at 58°C for 16 h to Illumina's Sentrix Mouse Ref-8v2 Expression BeadChips (Illumina, San Diego, CA). The arrays were washed, blocked and the labeled cRNA was detected by staining with streptavidin-Cy3. Hybridized arrays were scanned using an Illumina BeadStation 500× Genetic Analysis Systems scanner and the image data extracted using the Illumina GenomeStudio software, version 1.1.1.1. For statistical analysis, the expression data were filtered to include only probes with a consistent signal on each chip, and a detection *p *value of less than or equal to 0.02 for at least one sample of the data. The resulting dataset was next analyzed with DIANE 6.0, a spreadsheet-based microarray analysis program. An overview of DIANE can be found online at http://www.grc.nia.nih.gov/branches/rrb/dna/diane_software.pdf. Using DIANE, the results were normalized with a Z-Score transformation [50]. Z-normalized data were then analyzed with principal component analysis (PCA). To determine the gene expression changes within each specific RNA comparison, Z-Scores for paired treatment groups were compared using the Z-Ratio statistic [50]:

$$Z-Ratio=\frac{Z-Score_{LPS}-Z-Score_{Vehicle}}{\sigma \left[Z-Score_{LPS}-Z-Score_{Vehicle}\right]}$$

Expression changes for individual genes were considered significant if they met four criteria: Z-Ratio above 1.5 or below -1.5; false detection rate (FDR) [51] of less than 0.30; a *P*-value statistic for Z-Score replicability below 0.05; and mean background-corrected signal intensity greater than zero. Differentially expressed genes were identified as significant (*p *< 0.05) based on Z-scores. Gene set analysis using GO gene sets with the PAGE [52] algorithm was performed as previously described [53]. The data are publicly available at GEO (GSE31676).

### Immunoblot analysis

Whole cell extracts were prepared from Th2 cells by lysing cells in 50 mM Tris 7.4, 1% NP40, 150 mM NaCl, 0.5% Deoxycholate, 0.1% SDS and clearing the lysates by centrifugation. Protein extracts were separated on a 6% polyacrylamide gel and transferred to a PVDF membrane (BioRad). The immunoblots were blocked for 1 h at room temperature in 5% milk in TBST (50 mM Tris pH7.5, 100 mM NaCl, 0.03% Tween 20) and incubated with the BAF180 antibody (Bethyl A301-590A) or BRG1 antibody (Upstate/Millipore 07-478) overnight at 4°C. The blots were washed with TBST and incubated with anti-rabbit HRP-conjugated antibody (Zymed) at room temperature. After washing the blots with TBST, detection was carried out using enhanced chemiluminescence (Amersham) according to manufacturer's instructions.

### Chromatin immunoprecipitation (ChIP)

Chromatin immunoprecipitation was performed using methods similar to those described previously [21,24,47,54]; details are available on request. Approximately 20 million cells (for 3-5 immunoprecipitations) were crosslinked with 1% formaldehyde and quenched with glycine. Cells were lysed with buffer containing 1% SDS, treated with micrococcal nuclease, sonicated until the average DNA size was approximately 500 bp, and adjusted to 0.1% SDS, 1% Triton X-100 and 150 mM NaCl at 5 ml. Sonicates were precleared with protein A Sepharose (Upstate) and IP was performed with the following antibodies: 1 ug H3K9Ac (Abcam ab4441), 0.5 ul BRG1 (J1, Weidong Wang), 1 ug H3K18Ac (Abcam ab1191), 1 ug H3K4Me (Abcam ab8895), 2 ug BAF180 (A301-591A Bethyl Laboratories), 2 ug BAF250 (A301-041A Bethyl Laboratories), 1 ug CBP (Assay Biotech Ab-1535) or rabbit IgG (Santa Cruz sc-2027). Chromatin was collected with protein A, washed, eluted with TE pH 10.0 and crosslinks were reversed, followed by protease treatment. Chromatin was quantified by real-time PCR (Q-PCR) using an Applied Biosystems 7500 with Sybr Green detection (Biorad). Graphs indicate immunoprecipitated chromatin amounts relative to input DNA (% input). Oligo sequences are in Table 2.

**Table 2:** Primers for ChIP and DNase hypersensitivity

Locus	Primer name	Primer 1	Primer 2
IL-10-30.4	MP 1116	GCCCTTCTGGAGCTGGTTAGT	TCATACTTGGGCATGGAAATTTC

IL-10-29.8	MP 1117	GCTCTTGCTGCACATAATTCTGTAC	TGAAAGACTAGAACAAATGTGAACGA

IL-10-25.9	MP 2014	TCTGTTCCCAACTTAGGCTGC	GACCCACCAAAAGCTTCTGG

IL-10-23.3	MP 2015	CCTGGATGCGAAAGACCTCA	TGTGGATGGAGGGAGCATTC

IL-10-20.7	MP 1118	TGGATTGGCATGGGTAGAGAA	ATCACCCCAGACTGGATGTCA

IL-10-20.1	MP 1119	CCCTCCAGGTCTCGTCTCAAG	CTTTTGATTCCCATGCCTTACC

IL-10-17.2	MP 1120	CCTGCCTCATTATTAGCGTCTCTT	CATGGCCTTGGAAATAATATGCA

IL-10-17.0	MP 1121	TGAGAAGGTAAGAGGTTGCCATTA	TCTCTCCCCTGCCTCTTTTTC

IL-10-9	MP 911	AACACAGGTGAACACGCAAAAG	CTGGAAGTGCCATTCTGTAAGAGA

IL-10 pro	MP 913	GCCCATTTATCCACGTCATTATG	TGTTCTATGTACAGAGGCCCTCATC

IL-10 +1.8	MP 1246	GGTCTCTTGCTCATCTGTCTCTGA	AGGCTATGCGCAAATCTTCAC

IL-10 +3.2	MP 2016	CTCCCCCAAATCAGAACGAG	GCCCCGGGACAAGTAAGAAT

IL-10 +6.2	MP 2017	GCAGAGAGTGGGATGGCTCA	TCTCACTGGTGCCCGCA

IL-10 +18	MP 2018	AGGAGTTCAGGAGGCATGGA	TCACCATGTCTTGTGGTAACAGC

Nfm	MP 855	CCACGGCGCTGAAGGA	CTGGTGCATGTTCTGGTCTGA

## Results

### T lymphocyte development is essentially normal when the BAF180 gene is deleted relatively late in thymocyte development

Previous work from our laboratory identified a role for BRG-containing SWI/SNF complexes in the differentiation of Th2 cells as well as in the acute induction cytokine genes from Th2 and IL-3/GMCSF loci [24,35]. Additionally, using siRNA technology in effector Th cells we identified BAF250a-containing BAF complexes as an important SWI/SNF component in Th2 and IL-3/GMCSF cytokine expression [24,35]. By contrast, we did not identify a role for BAF180-containing PBAF complexes in effector Th2 cytokine expression and did not explore the role of BAF180 in Th2 differentiation. However, it remained possible that the siRNA-mediated partial depletion of BAF180 was not sufficient to reveal the role of BAF180 in T cells. To more thoroughly explore this issue we made use of a mouse strain made conditionally deficient for BAF180 in T cells [41]. Since we were testing the role of BAF180 in peripheral CD4 T cell function, we chose the CD4 promoter/enhancer driver for cre recombinase expression, as this expression cassette is expressed at a relatively late stage of thymocyte development [55]; we also expected T cell specific deletion would bypass the coronary and trophoblast defects occurring in embryos lacking BAF180 [40].

**Figure 1:**
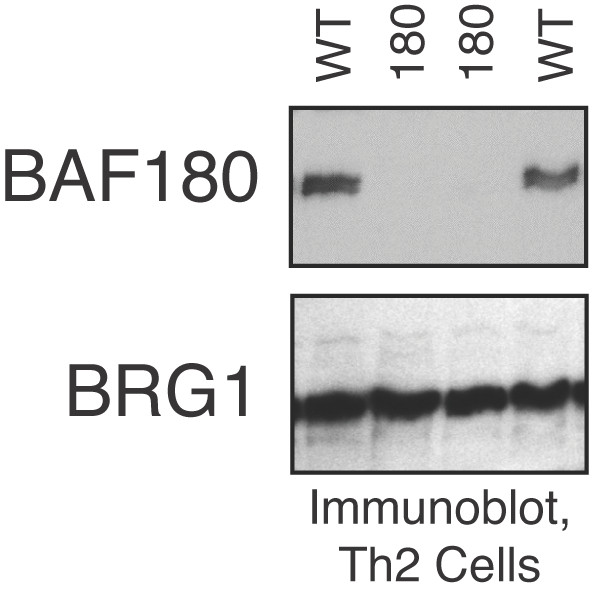
**T cell specific deletion of BAF180 Immunoblot of proteins from wild-type (WT) and BAF180-/- (180) Th2 cells**. BAF180 (upper panel) and BRG1 (lower panel) were visualized.

**Figure 2:**
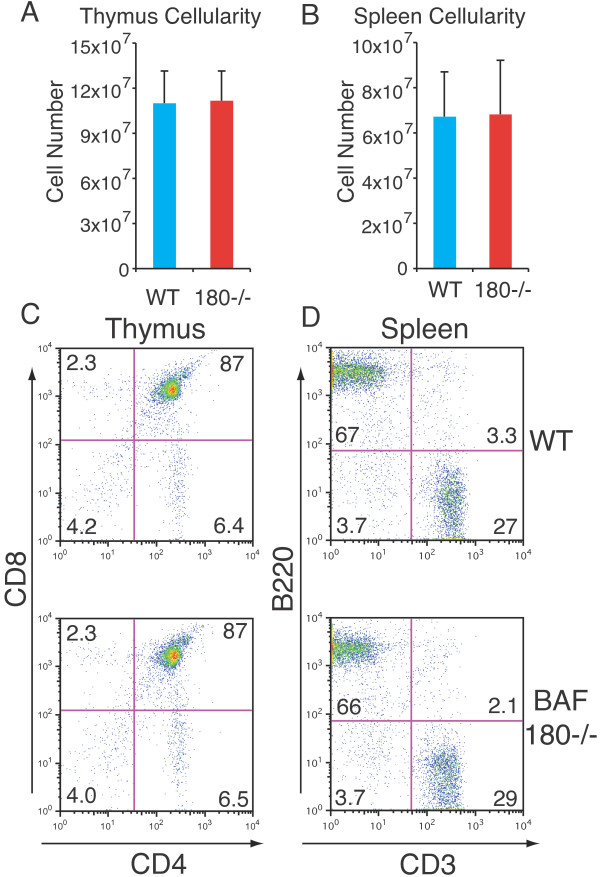
**T cell specific deletion of BAF180 has little or no effect on T cell development A,B) Cell numbers of A) thymus and B) spleen from wild-type (WT) and BAF180-/- (180-/-) mice, n = 5 pairs of mice**. C,D) Cell surface staining of C) CD8 and CD4 expression on thymocytes and D) B220 and CD3 expression on splenocytes from WT and BAF180-/- mice.

**Figure 3:**
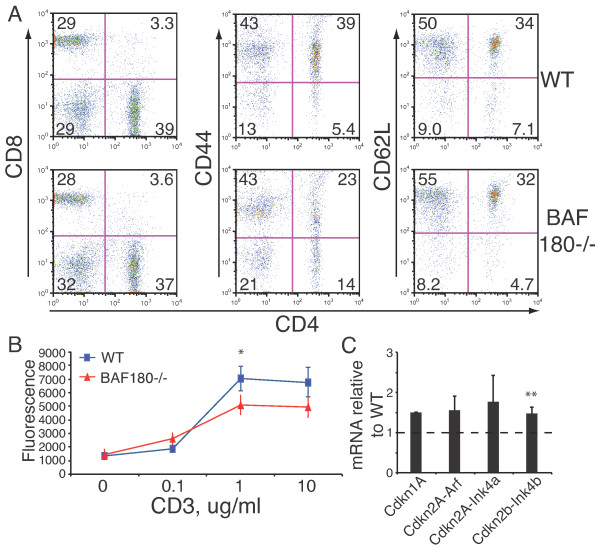
**Cell surface markers and proliferation of BAF180-deficient Th cells **A**) Cell surface staining of lymph nodes from WT (upper) and BAF180-/- (lower) mice, as a function of CD4 expression**. CD8 (left), CD44 (middle) and CD62L (right) were detected. **B**) Proliferation of CD4+ T cells purified from WT and BAF180-/- mice using the indicated concentration of plate bound anti-CD3 antibody and 1 μg/ml anti-CD28. Asterisk indicates *p *< 0.05. **C**) Cell cycle inhibitor RNA expression in restimulated Th2 cells from WT and BAF180-deficient mice was quantified by real time RT-PCR and normalized to WT values. n = 3. Double asterisk indicates *p *< 0.01; paired *T*-test values were Cdkn1A, 0.07; Cdkn2A-Arf, 0.21; Cdkn2A-Ink4a, 0.25, and Cdkn2B-Ink4B, 0.005. In stimulated naïve cells, the ratios/p values were Cdkn1A, 2.27/0.40; Cdkn2A-Arf, 3.34/0.02, Cdkn2A-Ink4a, 4.14/0.18, and Cdkn2B-Ink4B, 2.20/0.07.

As expected, in the presence of the cre recombinase transgene, we observed complete loss of BAF180 protein expression in T cells from BAF180fl/fl mice (Figure 1). By contrast, there was little if any effect on BRG1, another SWI/SNF component. Loss of BAF180 protein expression did not grossly affect cell number from the thymus, spleen or lymph nodes (Figure 2A, B and data not shown) suggesting that overall T cell development and expansion was not strongly affected by the loss of BAF180 at this late stage. This was further supported by FACS analysis showing typical staining profiles for CD4 and CD8 T cell subsets in both the thymus and lymphoid periphery, as well as typical T:B cell ratios in the spleen (Figures 2B, C, D and 3A).

We detected two subtle changes in BAF180-deficient peripheral CD4 T cells. First, although the naïve and central memory marker CD62L was unchanged in BAF180-deficient T cells, we observed a general down-regulation of the memory marker CD44 in peripheral T cells (Figure 3A). We do not know whether the loss of CD44 expression was a direct consequence of reduced CD44 transcription when BAF180 no longer functions on the CD44 gene, or alternatively an indirect effect of altered memory cell function in the absence of BAF180. In support of the former possibility, CD44 was one of the first bona fide SWI/SNF target genes identified in mammalian cells [56]. Additionally, we observed direct recruitment of PBAF complexes to multiple transcriptional elements in the CD44 gene (data not shown) suggesting that BAF180 was a direct positive regulator of CD44 expression in T cells. Second, BAF180-deficient CD4 T cells consistently displayed a slight decrease in proliferative response to T cell stimulation (Figure 3B). This correlated with a small increase in the expression of cell cycle inhibitors in BAF180-deficient T cells (Figure 3C).

### Enhanced Th2 differentiation in the absence of BAF180

**Figure 4:**
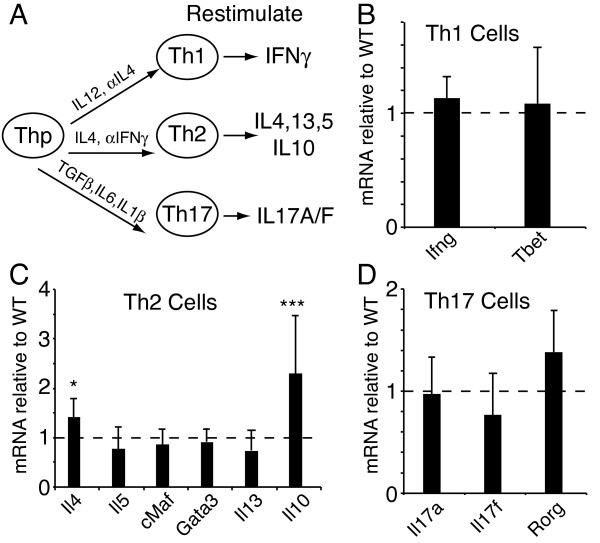
**Th differentiation in the absence of BAF180 A) Scheme for differentiation of T helper cells in culture, and restimulation with PMA/ionomycin**. B) Th1 cytokine and transcription factor expression as assessed by real time RT-PCR in WT and BAF180-/- stimulated Th1 cells. C) Th2 cytokine and transcription factor expression as assessed by real time RT-PCR comparing WT and BAF180-/- stimulated Th2 cells. D) Th17 cytokine and transcription factor expression as assessed by real time RT-PCR in WT and BAF180-/- stimulated Th17 cells. Results are the average and standard deviation of four independent experiments. The change in IL-10 was statistically significant (*p *< 0.005), the change in IL-4 was significant (*p *< 0.05) while none of the other tests here were significant.

In order to determine if BAF180 is involved in Th differentiation, we purified naïve CD4 T cells (Thp) from wildtype and BAF180-deficient mice and cultured the cells under Th1, Th2 and Th17 skewing conditions (Figure 4A); throughout this study, restimulated cells were activated with PMA and ionomycin. Analysis of expression of Th-subset specific cytokines and transcription factors revealed that, in the absence of BAF180, Th-differentiation was largely intact (Figure 4B, C, D). Additionally, BAF180-deficient T cells expanded similarly to wildtype T cells when cultured under skewing conditions with exogenous cytokines. We did consistently observe a slight enhancement of IL-4 expression and a larger increase in IL-10 expression in BAF180-deficient Th2 cells (Figure 4C). These results suggest that BAF180-deficient Th cells are largely capable of differentiating into several Th lineages and express Th cytokines appropriately. However, BAF180 appeared to repress IL-10 and perhaps IL-4 in Th2 cells.

### Deletion of BAF180 alters gene expression in Th2 cells

**Figure 5:**
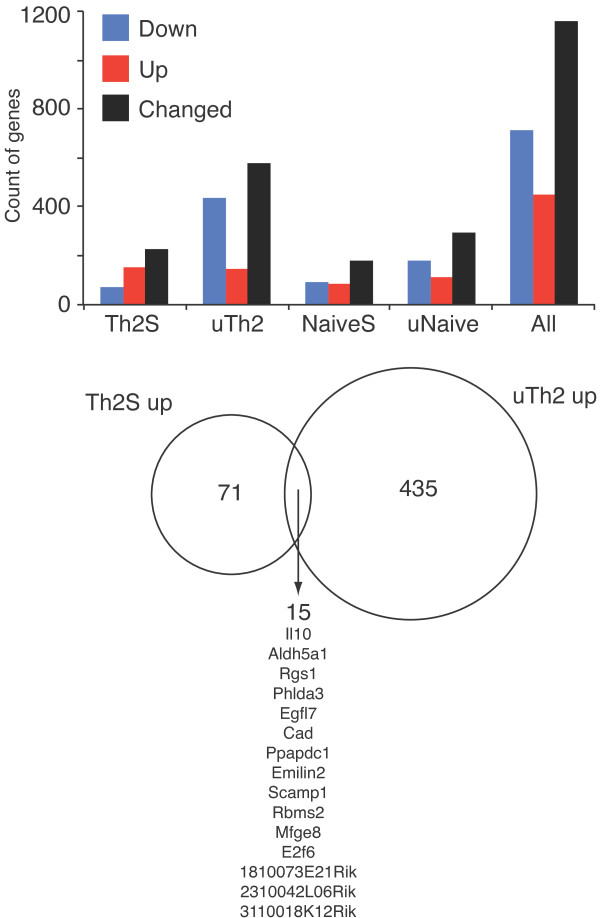
**BAF180**-**dependent gene expression changes in Th2 cells Upregulated and downregulated genes were counted in each cell type, after removing genes that were not expressed, and removing genes without statistically significant changes**. Total is the sum of upregulated and downregulated genes within a cell type. Cell types are listed below the graph; "Any" is a count of every gene that is regulated under at least one condition. Array data were derived from 3 pairs of WT and BAF180-/- mice. Data are deposited in GEO (GSE31676).

After observing some changes in candidate gene expression in BAF180-deficient Th2 cells, we expanded our analysis in a comprehensive, unbiased manner using beadchip analysis. We identified BAF180-dependent gene expression in resting and restimulated Th2 cells and Naïve CD4+ Th cells; primary data were deposited in GEO (GSE31676). We found the expression of approximately 1,100 genes was augmented or diminished in a statistically significant manner, following depletion of BAF180 protein in one or more of these conditions (Figure 5). More genes were affected in resting cells than in stimulated cells. In resting cells, more genes were upregulated than downregulated after deletion of BAF180, suggesting the major function of BAF180 in resting Th cells was to repress gene expression. More genes were affected in differentiated cells than in Naïve cells.

Among GO term gene sets in stimulated Th2 cells, decreased expression in pathways involving nucleosome assembly, transcription and DNA binding were evident in BAF180-deficient Th2 cells, while pathways involving ribosome biogenesis and translation were increased. Pathways altered in BAF180-/- Th2 cells involving immune cell function included "Positive Regulation of T cell Differentiation" and "Natural Killer Cell Activation". Genes that appeared to be repressed by BAF180 in Th2 cells included IL-10, Il2ra, Furin, Ctla4, Icos, Foxp3, Rgs1, Nras, E2f6, E2f1, Cdkn2a, Nfil3 and Jmjd1a. Genes that appeared to be activated by BAF180 in Th2 cells included Cd28, Stat1, Jak2, Twistnb, Daxx, Igf1r, Prkca, Chd4 Bcl2 Cdk2, Rb1, Ikbke, Egr1, and Adar. In Naïve cells, repressed genes included Cdkn1a, Foxp3, Gata3, Ifng, Il17f, Dnmt3b, while activated genes included Dicer1, Gadd45a, and Smad1. We note we had also identified Cdkn1a and Cdkn2a as repression targets using a candidate gene approach (Figure 3C). BAF180 repression targets in resting and stimulated Th2 cells were largely distinct; only a small number of targets were shared. Interestingly, the gene most enhanced by BAF180-deficiency in both resting and activated Th2 cells was IL-10 suggesting a role for BAF180 in the down-regulation of this cytokine.

### IL-10 expression in Th2 cells is negatively regulated by BAF180

**Figure 6:**
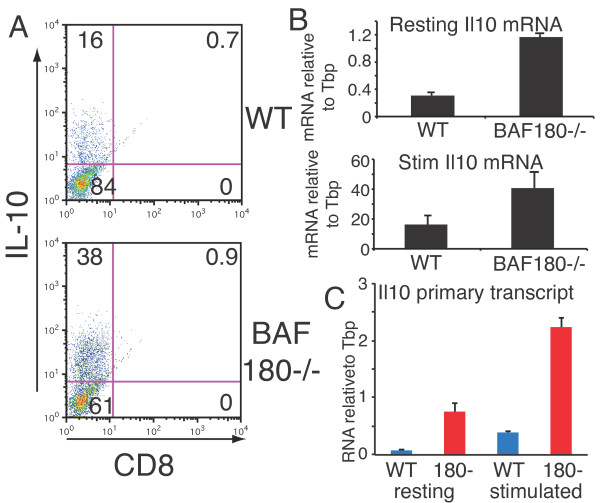
**BAF180 is a repressor of IL-10 transcription in Th2 cells A) Intracellular staining for IL-10 protein in WT and BAF180-/- Th2 cells**. B) IL-10 mRNA was quantified by real time RT-PCR from resting and activated Th2 cells. C) IL-10 primary transcripts were quantified by RT-PCR using primers specific to primary transcripts including intronic sequences from IL-10 locus. The results in B and C are the average and standard deviation of two independent experiments.

We validated the IL-10 expression changes in BAF180-deficient Th2 cells at both the protein and RNA level. Using intracellular cytokine staining, we observed that the number of IL-10 producing Th2 cells increased from 16% to 38% in the absence of BAF180 (Figure 6A). This correlated well with 2-4 fold enhanced IL-10 mRNA production in both resting and activated BAF180-deficient Th2 cells (Figure 6B). To determine if the enhanced IL-10 expression was due to increased transcription, we quantified the IL-10 primary (unspliced) transcript and similarly found an increase of at least 4 fold in resting and activated BAF180-/- cells (Figure 6C). (Incidentally, stimulation increased the IL-10 primary transcript 5 fold (Figure 6C) in WT cells, while the mRNA increased 50 fold (Figure 6B) and 10 fold (microarray experiment); while these differences may reflect experiment to experiment variation, they also provide some support for regulation of IL-10 mRNA stability.) These results demonstrate that the transcription of IL-10 is elevated in the absence of BAF180 and suggest that BAF180 is a repressor of IL-10 transcription.

### Enhanced BAF recruitment and histone acetylation at IL-10 locus in Th cells lacking BAF180

**Figure 7:**
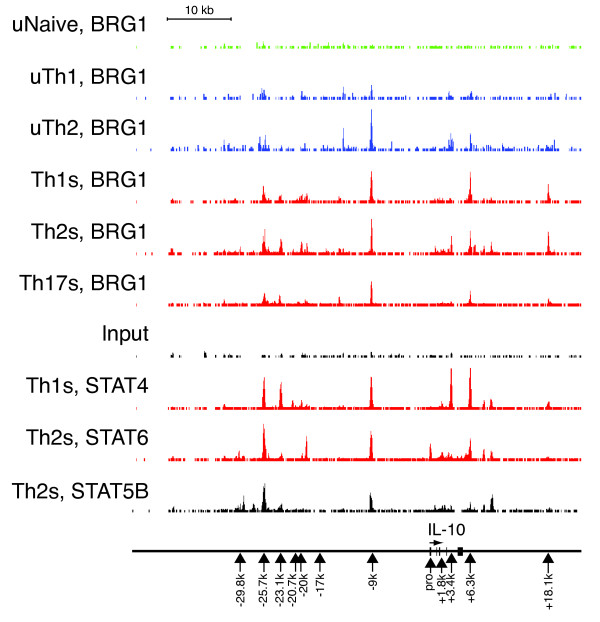
**BRG1 and transcription factor binding at the IL-10 locus in multiple T helper subtypes**. ChIP-seq profiles from T helper cells for BRG1, STAT6, STAT4 and STAT5B are shown. BRG1 data are from [47], Stat6 data are from [57] and Stat5 data are from [58]. Resting naïve cells (uNaive), resting Th1, (uTh1), resting Th2 (uTh2), re-stimulated Th1 (Th1s), re-stimulated Th2 (Th2s), re-stimulated Th17 (Th17s) cells are shown. Input is shown as a control. Occupancy range values (y axis) are identical for all graphs to allow direct comparison (minimum tag frequency of 0, maximum tag frequency of 1.14 × 10^-5^). A scale bar for the × axis (genomic location) is shown. IL-10 exons are indicated as vertical bars, and an arrow indicates the direction of transcription; vertical arrows indicate features analyzed in other figures, and their distance relative to the IL-10 promoter. The genomic coordinates represented (MM9 assembly) are chromosome 1, 132,870,000 to 132,940,000.

The IL-10 locus is marked by a number chromatin structure changes in Th2 cells in response to both activation and lineage-specific signals [6-8]. These modifications include histone acetylation, methylation and the generation of DNase I hypersensitive sites present over many kilobases upstream and downstream the IL-10 coding sequence. We examined the landscape of BRG1 binding and STAT transcription factor binding at this locus using ChIP-seq data. We found BRG1 at several regions near the IL-10 gene (Figure 7). Binding was strongest at regions upstream and downstream from the IL-10 promoter than at the promoter itself, as seen previously with cytokine genes [21,24,35,47]. More and stronger BRG1 binding was found in stimulated cells than in resting cells, and in Th2 fate than in other fates. BRG1 binding was statistically significant at -25.9 k, -23.4 k, -20 k, -9 k, +6.2 k, +9.6 k, and +18.5 k. Previous studies found DNase hypersensitivity at most of these sites [6-8]; BRG1 has previously been found to play a role in formation of DHS [24,35,47]. We note that STAT transcription factor binding overlaps with a number of these sites, and suggest that transcription-factor mediated recruitment might explain some of the observed BRG1 binding. Interestingly, STAT4 binding occurs in Th1 cells at locations lacking STAT6 in Th2 cells; these could be negatively acting sites.

**Figure 8:**
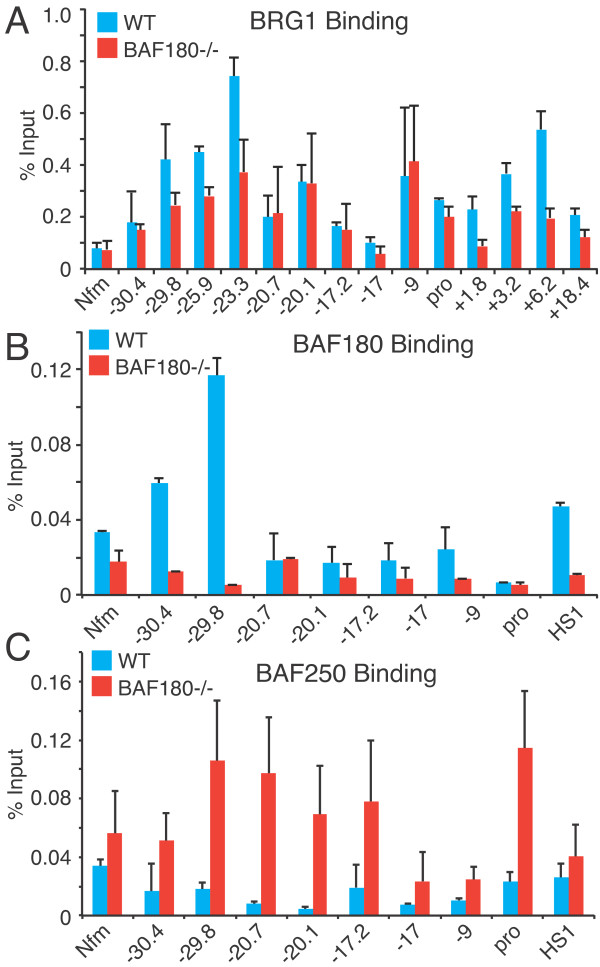
**Enhanced BAF250 recruitment to IL-10 locus in stimulated Th2 cells in the absence of BAF180 Binding of BRG1, BAF180 and BAF250 was detected by ChIP-PCR at the indicated sites in the IL-10 locus**. Signal is expressed as percent of the input chromatin. Control IP levels are less than 0.05% input. Nfm is a locus in the neuron-specific medium neurofilament gene, a binding site for BRG1 in neurons and brain but not in T cells. The results are the average and standard deviation of three ChIP experiments. A) BRG1 binding. B) BAF180 binding. C) BAF250 binding.

We examined BRG1, BAF180, and BAF250 binding at the IL-10 locus using ChIP-PCR. We found BRG1 binding at several regions, consistent with the ChIP-seq results (Figure 8A); BRG1 binding was strongest at -29.8 k, -25.9 k, -23.3 k, -9 k, and +6.2 k. BRG1 binding was low in naïve cells, induced during differentiation, decreased in resting cells, and was strongest in re-stimulated cells (data not shown), as seen with the Th2 cytokine loci [24]. We found BRG1 was required during Th2 differentiation to establish DHS at -9 k and +1.8 k (data not shown), as found previously at the IL-4 and GATA3 promoters [24]. The binding of the BRG1 paralog Brm was similar to BRG1 (data not shown). BRG1 binding was not affected by BAF180 deletion at some sites, such as -9 k, while other sites such as -29.8 k BRG1 and the above-mentioned STAT4 binding sites in Th1 cells, BRG1 binding was reduced. We next examined BAF180 binding to the IL-10 locus; we detected BAF180 binding especially at distal sites (-30.4 k, -29.8 k) and the IL-10 promoter in Th2 cells (Figure 8B). The pattern of BAF180 binding was similar to BRG1, with the prominent exception that little if any BAF180 binding occurred at -9 k, a strong BRG1 binding site. As expected, BAF180 binding was absent in BAF180-deficient Th2 cells (Figure 8B). Finally we measured BAF250a binding as a marker of BAF complexes. We detected enhanced binding of BAF250a to distal elements and the IL-10 promoter in BAF180-deficient Th2 cells compared to WT cells (Figure 8C). The strongest BAF250a binding was to the IL-10 promoter, a weak BRG1 site, while there was little if any BAF250a binding to -9 k, a strong BRG1 site. These results suggest that while overall SWI/SNF recruitment is not strongly affected by BAF180-deficiency, the composition of the SWI/SNF complex (BAF vs PBAF) is altered and thus changing the composition of SWI/SNF changes IL-10 gene expression.

**Figure 9:**
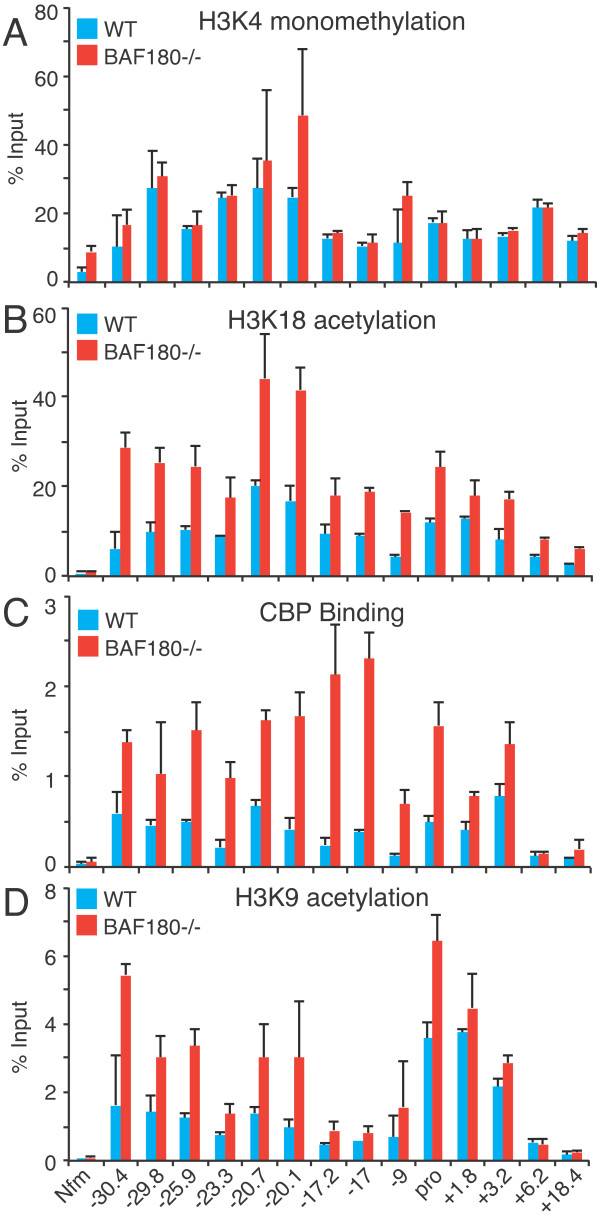
**Enhanced histone acetylation and CBP recruitment to IL-10 locus in BAF180-deficient Th2 cells Histone modifications and binding of the histone acetyltransferase CBP were detected by ChIP-PCR at the indicated sites in the IL-10 locus**. Signal is expressed as percent of the input chromatin. Control IP levels are less than 0.05% input. Nfm is a locus in the neuron-specific medium neurofilament gene, a binding site for BRG1 in neurons and brain but not in T cells. The results are the average and standard deviation of three ChIP experiments. A) Histone H3K4 monomethylation. B) Histone H3K18 acetylation. C) CBP binding. D) Histone H3K9 acetylation.

Next, we examined histone modifications and modifiers at the IL-10 locus using ChIP-PCR, as we had previously found BRG1 regulated histone acetylation [24]. H3K4me1, a mark frequently found at enhancers, was found at all the tested regions of the IL-10 locus, especially at -29.8 k, -20.7 k, -20.1 k, and -9 k (Figure 9A, blue bars). H3K4me1 was not strongly affected by deletion of BAF180 (red bars), though IL-10 expression was enhanced in BAF180-deficient Th2 cells. H3K18ac and H3K9ac, marks that are enriched at active enhancers and promoters [59], were found at all the tested regions of the IL-10 locus; H3K18ac was especially prominent at -20.7 k and -20.1 k, H3K9ac especially at the promoter and downstream regions (blue bars). Histone acetylation was elevated in BAF180-deficient cells (Figure 9B and 9D, red bars); this difference was prominent at sites distal to the IL-10 promoter (-30.4 k, -29.8 k, -20.7 k, -20.1 k). These results suggested that the replacement of PBAF complexes with BAF complexes altered histone modifications of the locus, or alternatively that these histone modifications were acquired as a consequence of SWI/SNF-mediated transcriptional regulation. The CBP and p300 histone acetyltransferases been shown experimentally to generate the H3K18ac and H3K27ac modification [60,61] associated with active enhancers and promoters [59,62-65], and these paralogs bind similar targets [66]. We found CBP binding to many of the tested regions in the IL-10 locus; we detected increased CBP recruitment to IL-10 enhancers and the IL-10 promoter in the absence of BAF180 (Figure 9C). Therefore, the composition of specific SWI/SNF complexes can influence histone acetyltransferase recruitment and histone acetylation; as the changes in histone modifications do not always coincide with changes in BAF180 binding, we do not know whether they are the result of looping, or spreading of histone modifications. These alterations in histone modifications might be programmed by SWI/SNF to direct transcription, or might be a consequence of transcription programmed by SWI/SNF.

### Binding of BRG1 and transcription factors in the IL24/IL20/IL19/IL10 locus

**Figure 10:**
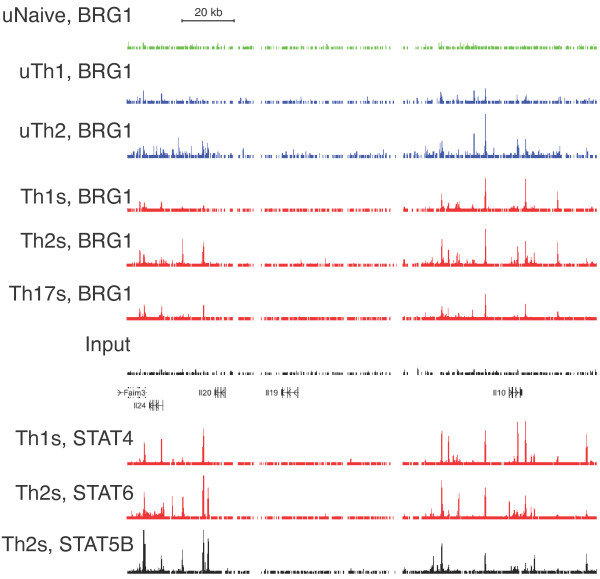
**BRG1 Binding at the IL-10/IL-24 Locus in multiple T helper subtypes**. ChIP-seq profiles from T helper cells for BRG1, STAT6, STAT4 and STAT5B are shown. BRG1 data are from [47], Stat6 data are from [57] and Stat5 data are from [58]. Resting naïve cells (uNaive), resting Th1, (uTh1), resting Th2 (uTh2), re-stimulated Th1 (Th1s), re-stimulated Th2 (Th2s), re-stimulated Th17 (Th17s) cells are shown. Input is shown as a control. Occupancy range values (y axis) are identical for all graphs to allow direct comparison (minimum tag frequency of 0, maximum tag frequency of 1.14 × 10^-5^). A scale bar for the × axis (genomic location) is shown. Exons are indicated as vertical bars, gene names are to the left, and arrowheads indicate the direction of transcription. The genomic coordinates represented (MM9 assembly) are chromosome 1, 132,770,000 to 132,950,000.

IL-10 is part of a multi-gene cluster in mouse and human. A recent report indicated that IL-24 expression is Th2 specific, mediated in part by STAT6 function through the IL-24 promoter [67]. We asked whether there might be functional elements dispersed throughout this locus, as found for the IL4/IL13/IL5 locus; these can be identified through genomic analysis, especially when multiple datasets are combined [4,68]. We found BRG1 binding clustered around the IL-24 and IL-10 genes (Figure 10). There were few if any binding regions in the central 60 kb interval containing the IL-20 and IL-19 genes. In resting and stimulated cell types, BRG1 binding was Th2 specific, and binding was stronger in stimulated cells. Consistent with our previous global analysis and analysis of specific genes, these effects were not absolute; for example, there is substantial BRG1 binding in Th1 cells; it is not clear whether this is the result of combinatorial control, or if these sites can be both positively and negatively acting. STAT6 and STAT5 binding was present at numerous upstream and downstream regions, extending the observation of STAT6 at the IL-24 promoter. The location of statistically significant binding regions for both loci is presented as a table (See Additional file 1) of genomic coordinates and features, organized by factor/condition (By Factor tab) and by genomic coordinate (By site tab). We note that occupancy of the IL-19 -19.8 k and IL-10 +3.2 k sites was detected in cells that do not express these genes under these conditions; perhaps negative regulatory elements lie in these regions. We also note that the IL-10 -25.9 k, IL-10 -9 k, IL-10 +9.5 k, and IL-20 +8.6 k elements are bound by BRG1, STAT6, STAT5A and STAT5B; perhaps these are especially important positive elements. We have confirmed that these elements are DHS in Th2 cells (data not shown).

## Discussion

We examined the role of ATP-dependent chromatin remodeling in T cell function. Our previous work identified an activating role for BAF complexes, SWI/SNF complexes containing BAF250, in cytokine gene expression in T helper cells [24,35,47]. In the current study we examined the contribution of PBAF, a different SWI/SNF complex containing BAF180, in T cell function and cytokine expression using T cell specific BAF180-deficient mice. We identified numerous BAF180-dependent changes in gene expression in our microarray analysis; however, overall Th cell development and cytokine expression were intact. BAF180 was a negative regulator of IL-10 transcription and, in the absence of BAF180, histone modifications were reprogrammed and BAF250-containing BAF complexes were recruited to the IL-10 locus.

We find the binding patterns of BAF180 and BAF250 are overlapping, yet readily distinguishable. Our data are consistent with the model that SWI/SNF exists in distinct BAF and PBAF complexes, determined by several independent research groups examining different species. Our data are also consistent with a stepwise assembly model, as proposed based upon time-resolved cell imaging studies [69]. These data are more difficult to reconcile with the proposal that BAF180 and BAF250 are, or can be, in the same complex [70,71]. We note that genetic studies have found different targets and functions for the BAF-specific subunits BAF250a/Arid1a and BAF250b/Arid1b in ES cells [36,37]. Differences have also been found for the PBAF-specific subunits BAF180, BAF200 and BRD7 [25,31]. There may be different BAF and PBAF complexes; alternatively, it may be that only some SWI/SNF subunits within a complex contribute to function at a particular locus. Direct, comprehensive comparison of these subunits would extend our understanding of the SWI/SNF system.

The targeting of remodeling complexes to specific DNA elements is mediated by association with transcription factors, histone modifications, non-coding RNA and interactions with the general transcription machinery [4,42,72-76]. Our previous studies identified important roles for transcription factors, particularly STATs, in the recruitment of BRG1 to specific regulatory elements in T cells [4,24,47]. Interestingly, Stat6 has been shown to be a critical transcription factor in the Th2-specific expression of IL-10 and IL-24 [67,77], and analysis of BRG1 and Stat6 ChIP-Seq data reveal Stat6 binding at many of the distal regulatory elements in the IL-10 locus occupied by BAF and PBAF complexes [47,57]. Whether or not Stat6 is mediating the recruitment of SWI/SNF to the IL-10 locus or if Stat6 preferentially interacts with BAF or PBAF complexes remains to be determined. Ets-1 is reported to be a repressor of IL-10 expression [78,79], and Ets sequence motifs are enriched at BRG1 binding sites in resting Th cells [47].

Our recent examination of BRG1 did not reveal repression as a prominent function for SWI/SNF, by comparison of Th2S gene expression and BRG1 binding [47]. We note that most BAF180-mediated repression was in uTh2 cells, and we did not examine resting cell gene expression in our previous work. Alternatively, there may be more SWI/SNF activation targets than repression targets; this assessment awaits analysis of BAF250a KO and BRG1 KO Th2 cells for direct comparison under identical conditions. A previous report suggested Brm more important for repression, BRG1 more important for activation [80]. It is unclear how BAF180 complexes are mediating repression; they may be working as repressor complexes, or preventing the binding and function of activator complexes containing BAF250.

It is perhaps surprising that we found that deletion of BAF180 (late in T cell development) does not strongly perturb early T cell development. We have used a CRE expression cassette that induces deletion at a late DN stage, potentially bypassing an earlier requirement for BAF180 expression. Several other factors, such as GATA3, BRG1, TCF7, β-catenin and BPTF, appear to be required for early T cell development [22,81-85]. Given that BRG1 has obvious effects on T cell development, we could have found that BAF180 deletion would also have strong effects on T cell development. BAF180-deficient mice have defects in cardiac development [41] and BAF180 is a cell cycle regulator in some settings [43,44]; it is not clear what determines the relative importance of PBAF complexes in different cell states.

Genetic variants within IL-10 have been associated with human disease. The human SNP rs3024505, associated with Type 1 Diabetes, Crohn's disease and ulcerative colitis [15-17], maps to BRG1, STAT6, and STAT4 binding at +6.3 k in the mouse. Ulcerative colitis is also linked to rs3024493 [16], within intron 3; this is 1 k upstream of BRG1, STAT4 and CBP binding at +3.2 k, and slightly downstream of BRG1 binding and H3K9 acetylation at +1.8 k. Behcet's disease is associated with a variant (rs1800871) that lies near the IL-10 promoter [18], adjacent to BRG1, STAT6 and CBP binding. The proximity of these genetic variants to these remodeling enzyme and transcription factor binding regions suggests these binding regions may regulate IL-10 gene expression in a physiologically relevant manner.

Previous studies on breast cancer cells suggested BAF180 is a tumor suppressor gene that positively regulates the expression of the cell cycle inhibitor p21 through direct interaction and activation of the p21 promoter [44]. A positive role for BAF250a in the regulation of p21 transcription has been suggested in studies in a pre-osteoblast line [38]. However BAF250a can also serve as a repressor of c-myc in these same cells while another BAF-specific protein, BAF250b, is an activator of c-myc [38]. We observed a slight decrease in the ability of BAF180-deficient Th cells to proliferate in response to T cell activation and enhanced expression of cell cycle inhibitors, including p21 (CDKN1), suggesting that in T cells BAF180 is a repressor of these genes. We also observed binding of PBAF complexes to the p21 locus in T cells (data not shown). At this time, there is no simple relationship between BAF/PBAF complexes and cell cycle progression.

## Conclusions

The relative contribution of different SWI/SNF subtypes in T cell gene expression is largely unknown. Here we identify gene targets in Th2 cells regulated by the PBAF-specific SWI/SNF component, BAF180. In particular, we find that BAF180 is a repressor of IL-10 gene transcription. BAF180 binds directly to regulatory elements in the IL-10 locus but is replaced by the BAF-specific component, BAF250a, in the absence of BAF180, resulting in enhanced histone acetylation at the IL-10 locus. This study demonstrates that the differential recruitment of SWI/SNF subtypes can have direct consequences on chromatin structure and gene transcription.

## Competing interests

The authors declare that they have no competing interests.

## Authors' contributions

The authors have made the following declaration about their contributions: Conceived and designed the experiments: ALW, MJP, ZW Performed the experiments:, ALW, PP, WHW, MJP, Analyzed the data, ALW, YZ, KGB, MJP, Wrote the paper ALW, MJP. All authors read and approved the final manuscript.

## Supplementary Material

Additional file 1 **IL -10/IL-19/IL-20/IL-24 locus features are described in the excel file < IL10_IL24_Features.xlsx> On the tab "By Factor", each row reports a feature in the locus identified by ChIP-seq**. This presentation facilitates identifying primer sequences for future studies. Columns A, B, and C report the mm9 chromosomal coordinates, column D, the length of the feature in bp, column E, the nearest transcriptional start site (TSS), Columns E, F, G, H, and I, the Gene Symbol ref-seq accession number, TSS, and transcription end site (TSE) for the gene containing the nearest TSS, Column J, the distance from the center of the feature to the nearest TSS, in bp; and Column K, condition, the feature present, when reported as statistically significant, using Cis Genome [86]; uN_BRG1 indicates BRG1 binding in resting Naïve cells, uN_BRG1 indicates BRG1 binding in unstimulated Naïve cells, u2_BRG1 indicates BRG1 binding in unstimulated Th2 cells, 1S_BRG1 indicates BRG1 binding in Stimulated Th1 cells, 2S_BRG1 indicates BRG1 binding in Stimulated Th2 cells, 17S_BRG1 indicates BRG1 binding in Stimulated Th17 cells, 2S_H3K4me3 indicates H3K4 trimethylation, a mark frequently found slightly downstream of active TSS, 2S_STAT6 indicates STAT6 binding in stimulated Th2 cells,, 2S_STAT5A, 2S_STAT5B indicate binding of STAT5A and STAT5B, respectively, in stimulated Th2 cells, 1S_STAT4 indicates binding of STAT4 in stimulated Th1 cells. BRG1 data are from [47], STAT6 and STAT4 data are from [57], STAT5 data are from [58], H3K4me3 data are from [87]. On the tab "By Site" each row is a chromosomal location, and the columns indicate location and features. This presentation facilitates identifying which features are present at the same site, and how the features change within the T helper subsets. Columns A and B indicate the Gene Symbol and approximate distance to the nearest TSS; Columns D-N tabulate the features above, in lineage order; Yes in any box indicates the feature identified in that column is present in the location described in that row; present meaning identified as statistically significant using CisGenome. Column N, GWAS findings, indicates locations that are homologous to human regions (or nearby homologous regions) that contain genetic variants that are in linkage disequilibrium with the indicated conditions, as described in the discussion. The "By Site" tab was prepared using the data from the "By Factor" tab. Note some locations, such as IL-10 -25.8 k, -9 k, +6.2 k, and +9.5 k, IL-20 +8.7 k, IL-24 +9 k and +7.4 k, are annotated for statistically significant findings under several conditions/features. Click here for file

## References

[B1] Lee GR Kim ST Spilianakis CG Fields PE Flavell RA T helper cell differentiation: regulation by cis elements and epigenetics Immunity 2006 24 4 369 379 10.1016/j.immuni.2006.03.007 16618596

[B2] Ansel KM Djuretic I Tanasa B Rao A Regulation of Th2 differentiation and Il4 locus accessibility Annu Rev Immunol 2006 24 607 656 10.1146/annurev.immunol.23.021704.115821 16551261

[B3] Wilson CB Rowell E Sekimata M Epigenetic control of T-helper-cell differentiation Nat Rev Immunol 2009 9 2 91 105 10.1038/nri2487 19151746

[B4] Wurster AL Pazin MJ ATP-dependent chromatin remodeling in T cells Biochemistry and cell biology = Biochimie et biologie cellulaire 2011 10.1139/o11-042 10.1139/o11-042 PMC3424609 21999456

[B5] Saraiva M O'Garra A The regulation of IL-10 production by immune cells Nat Rev Immunol 2010 10 3 170 181 10.1038/nri2711 20154735

[B6] Jones EA Flavell RA Distal enhancer elements transcribe intergenic RNA in the IL-10 family gene cluster J Immunol 2005 175 11 7437 7446 16301651 10.4049/jimmunol.175.11.7437

[B7] Kang KH Im SH Differential regulation of the IL-10 gene in Th1 and Th2 T cells Ann N Y Acad Sci 2005 1050 97 107 10.1196/annals.1313.011 16014524

[B8] Im SH Hueber A Monticelli S Kang KH Rao A Chromatin-level regulation of the IL10 gene in T cells J Biol Chem 2004 279 45 46818 46825 10.1074/jbc.M401722200 15319439

[B9] Vieira P O'Garra A Regula'ten' the gut Nat Immunol 2007 8 9 905 907 10.1038/ni0907-905 17712340

[B10] Veldhoen M Uyttenhove C van Snick J Helmby H Westendorf A Buer J Martin B Wilhelm C Stockinger B Transforming growth factor-beta 'reprograms' the differentiation of T helper 2 cells and promotes an interleukin 9-producing subset Nat Immunol 2008 9 12 1341 1346 10.1038/ni.1659 18931678

[B11] Saraiva M Christensen JR Veldhoen M Murphy TL Murphy KM O'Garra A Interleukin-10 production by Th1 cells requires interleukin-12-induced STAT4 transcription factor and ERK MAP kinase activation by high antigen dose Immunity 2009 31 2 209 219 10.1016/j.immuni.2009.05.012 19646904 PMC2791889

[B12] Kuhn R Lohler J Rennick D Rajewsky K Muller W Interleukin-10-deficient mice develop chronic enterocolitis Cell 1993 75 2 263 274 10.1016/0092-8674(93)80068-P 8402911

[B13] Hawrylowicz CM O'Garra A Potential role of interleukin-10-secreting regulatory T cells in allergy and asthma Nat Rev Immunol 2005 5 4 271 283 10.1038/nri1589 15775993

[B14] Shevach EM Mechanisms of foxp3+ T regulatory cell-mediated suppression Immunity 2009 30 5 636 645 10.1016/j.immuni.2009.04.010 19464986

[B15] Barrett JC Clayton DG Concannon P Akolkar B Cooper JD Erlich HA Julier C Morahan G Nerup J Nierras C Genome-wide association study and meta-analysis find that over 40 loci affect risk of type 1 diabetes Nat Genet 2009 41 6 703 707 10.1038/ng.381 19430480 PMC2889014

[B16] Franke A McGovern DP Barrett JC Wang K Radford-Smith GL Ahmad T Lees CW Balschun T Lee J Roberts R Genome-wide meta-analysis increases to 71 the number of confirmed Crohn's disease susceptibility loci Nat Genet 2010 42 12 1118 1125 10.1038/ng.717 21102463 PMC3299551

[B17] McGovern DP Gardet A Torkvist L Goyette P Essers J Taylor KD Neale BM Ong RT Lagace C Li C Genome-wide association identifies multiple ulcerative colitis susceptibility loci Nat Genet 2010 42 4 332 337 10.1038/ng.549 20228799 PMC3087600

[B18] Mizuki N Meguro A Ota M Ohno S Shiota T Kawagoe T Ito N Kera J Okada E Yatsu K Genome-wide association studies identify IL23R-IL12RB2 and IL10 as Behcet's disease susceptibility loci Nat Genet 2010 42 8 703 706 10.1038/ng.624 20622879

[B19] Flaus A Martin DM Barton GJ Owen-Hughes T Identification of multiple distinct Snf2 subfamilies with conserved structural motifs Nucleic Acids Res 2006 34 10 2887 2905 10.1093/nar/gkl295 16738128 PMC1474054

[B20] Saha A Wittmeyer J Cairns BR Chromatin remodelling: the industrial revolution of DNA around histones Nat Rev Mol Cell Biol 2006 7 6 437 447 10.1038/nrm1945 16723979

[B21] Precht P Wurster AL Pazin MJ The SNF2H chromatin remodeling enzyme has opposing effects on cytokine gene expression Mol Immunol 2010 47 11-12 2038 2046 10.1016/j.molimm.2010.04.009 20471682 PMC2891439

[B22] Landry JW Banerjee S Taylor B Aplan PD Singer A Wu C Chromatin remodeling complex NURF regulates thymocyte maturation Genes Dev 2011 25 3 275 286 10.1101/gad.2007311 21289071 PMC3034902

[B23] Ramirez J Hagman J The Mi-2/NuRD complex: a critical epigenetic regulator of hematopoietic development, differentiation and cancer Epigenetics 2009 4 8 532 536 10.4161/epi.4.8.10108 19923891

[B24] Wurster AL Pazin MJ BRG1-mediated chromatin remodeling regulates differentiation and gene expression of T helper cells Mol Cell Biol 2008 28 24 7274 7285 10.1128/MCB.00835-08 18852284 PMC2593447

[B25] Kaeser MD Aslanian A Dong MQ Yates JR Emerson BM BRD7, a novel PBAF-specific SWI/SNF subunit, is required for target gene activation and repression in embryonic stem cells J Biol Chem 2008 283 47 32254 32263 10.1074/jbc.M806061200 18809673 PMC2583284

[B26] Chi T A BAF-centred view of the immune system Nat Rev Immunol 2004 4 12 965 977 10.1038/nri1501 15573131

[B27] Wang W Cote J Xue Y Zhou S Khavari PA Biggar SR Muchardt C Kalpana GV Goff SP Yaniv M Purification and biochemical heterogeneity of the mammalian SWI-SNF complex EMBO J 1996 15 19 5370 5382 8895581 PMC452280

[B28] Wang W Xue Y Zhou S Kuo A Cairns BR Crabtree GR Diversity and specialization of mammalian SWI/SNF complexes Genes Dev 1996 10 17 2117 2130 10.1101/gad.10.17.2117 8804307

[B29] Lemon B Inouye C King DS Tjian R Selectivity of chromatin-remodelling cofactors for ligand-activated transcription Nature 2001 414 6866 924 928 10.1038/414924a 11780067

[B30] Nie Z Xue Y Yang D Zhou S Deroo BJ Archer TK Wang W A specificity and targeting subunit of a human SWI/SNF family-related chromatin-remodeling complex Mol Cell Biol 2000 20 23 8879 8888 10.1128/MCB.20.23.8879-8888.2000 11073988 PMC86543

[B31] Yan Z Cui K Murray DM Ling C Xue Y Gerstein A Parsons R Zhao K Wang W PBAF chromatin-remodeling complex requires a novel specificity subunit, BAF200, to regulate expression of selective interferon-responsive genes Genes Dev 2005 19 14 1662 1667 10.1101/gad.1323805 15985610 PMC1176002

[B32] Moshkin YM Mohrmann L van Ijcken WF Verrijzer CP Functional differentiation of SWI/SNF remodelers in transcription and cell cycle control Mol Cell Biol 2007 27 2 651 661 10.1128/MCB.01257-06 17101803 PMC1800805

[B33] Lessard J Wu JI Ranish JA Wan M Winslow MM Staahl BT Wu H Aebersold R Graef IA Crabtree GR An essential switch in subunit composition of a chromatin remodeling complex during neural development Neuron 2007 55 2 201 215 10.1016/j.neuron.2007.06.019 17640523 PMC2674110

[B34] Ho L Ronan JL Wu J Staahl BT Chen L Kuo A Lessard J Nesvizhskii AI Ranish J Crabtree GR An embryonic stem cell chromatin remodeling complex, esBAF, is essential for embryonic stem cell self-renewal and pluripotency Proc Natl Acad Sci USA 2009 106 13 5181 5186 10.1073/pnas.0812889106 19279220 PMC2654396

[B35] Wurster AL Precht P Pazin MJ NF-kappaB and BRG1 bind a distal regulatory element in the IL-3/GM-CSF locus Molecular immunology 2011 48 15-15 2178 2188 21831442 10.1016/j.molimm.2011.07.016 PMC3163777

[B36] Gao X Tate P Hu P Tjian R Skarnes WC Wang Z ES cell pluripotency and germ-layer formation require the SWI/SNF chromatin remodeling component BAF250a Proc Natl Acad Sci USA 2008 105 18 6656 6661 10.1073/pnas.0801802105 18448678 PMC2373334

[B37] Yan Z Wang Z Sharova L Sharov AA Ling C Piao Y Aiba K Matoba R Wang W Ko MS BAF250B-associated SWI/SNF chromatin-remodeling complex is required to maintain undifferentiated mouse embryonic stem cells Stem Cells 2008 26 5 1155 1165 10.1634/stemcells.2007-0846 18323406 PMC2409195

[B38] Nagl NG Jr Wang X Patsialou A Van Scoy M Moran E Distinct mammalian SWI/SNF chromatin remodeling complexes with opposing roles in cell-cycle control EMBO J 2007 26 3 752 763 10.1038/sj.emboj.7601541 17255939 PMC1794396

[B39] Gui Y Guo G Huang Y Hu X Tang A Gao S Wu R Chen C Li X Zhou L Frequent mutations of chromatin remodeling genes in transitional cell carcinoma of the bladder Nat Genet 2011 43 9 875 878 10.1038/ng.907 21822268 PMC5373841

[B40] Wang Z Zhai W Richardson JA Olson EN Meneses JJ Firpo MT Kang C Skarnes WC Tjian R Polybromo protein BAF180 functions in mammalian cardiac chamber maturation Genes Dev 2004 18 24 3106 3116 10.1101/gad.1238104 15601824 PMC535920

[B41] Huang X Gao X Diaz-Trelles R Ruiz-Lozano P Wang Z Coronary development is regulated by ATP-dependent SWI/SNF chromatin remodeling component BAF180 Dev Biol 2008 319 2 258 266 10.1016/j.ydbio.2008.04.020 18508041

[B42] Thompson M Polybromo-1: the chromatin targeting subunit of the PBAF complex Biochimie 2009 91 3 309 319 10.1016/j.biochi.2008.10.019 19084573 PMC2646799

[B43] Burrows AE Smogorzewska A Elledge SJ Polybromo-associated BRG1-associated factor components BRD7 and BAF180 are critical regulators of p53 required for induction of replicative senescence Proc Natl Acad Sci USA 2010 107 32 14280 14285 10.1073/pnas.1009559107 20660729 PMC2922604

[B44] Xia W Nagase S Montia AG Kalachikov SM Keniry M Su T Memeo L Hibshoosh H Parsons R BAF180 is a critical regulator of p21 induction and a tumor suppressor mutated in breast cancer Cancer Res 2008 68 6 1667 1674 10.1158/0008-5472.CAN-07-5276 18339845 PMC2915562

[B45] Varela I Tarpey P Raine K Huang D Ong CK Stephens P Davies H Jones D Lin ML Teague J Exome sequencing identifies frequent mutation of the SWI/SNF complex gene PBRM1 in renal carcinoma Nature 2011 469 7331 539 542 10.1038/nature09639 21248752 PMC3030920

[B46] Li M Zhao H Zhang X Wood LD Anders RA Choti MA Pawlik TM Daniel HD Kannangai R Offerhaus GJ Inactivating mutations of the chromatin remodeling gene ARID2 in hepatocellular carcinoma Nat Genet 2011 43 9 828 829 10.1038/ng.903 21822264 PMC3163746

[B47] De S Wurster AL Precht P Wood WH Becker KG Pazin MJ Dynamic BRG1 recruitment during T helper differentiation and activation reveals distal regulatory elements Mol Cell Biol 2011 31 7 1512 1527 10.1128/MCB.00920-10 21262765 PMC3135292

[B48] Jung M Ramankulov A Roigas J Johannsen M Ringsdorf M Kristiansen G Jung K In search of suitable reference genes for gene expression studies of human renal cell carcinoma by real-time PCR BMC Mol Biol 2007 8 47 10.1186/1471-2199-8-47 17559644 PMC1913536

[B49] Potts RC Zhang P Wurster AL Precht P Mughal MR Wood WH Zhang Y Becker KG Mattson MP Pazin MJ CHD5, a Brain-Specific Paralog of Mi2 Chromatin Remodeling Enzymes, Regulates Expression of Neuronal Genes PLoS One 2011 6 9 e24515 10.1371/journal.pone.0024515 21931736 PMC3172237

[B50] Cheadle C Vawter MP Freed WJ Becker KG Analysis of microarray data using Z score transformation J Mol Diagn 2003 5 2 73 81 10.1016/S1525-1578(10)60455-2 12707371 PMC1907322

[B51] Tusher VG Tibshirani R Chu G Significance analysis of microarrays applied to the ionizing radiation response Proc Natl Acad Sci USA 2001 98 9 5116 5121 10.1073/pnas.091062498 11309499 PMC33173

[B52] Kim SY Volsky DJ PAGE: parametric analysis of gene set enrichment BMC Bioinforma 2005 6 144 10.1186/1471-2105-6-144 PMC1183189 15941488

[B53] De S Zhang Y Garner JR Wang SA Becker KG Disease and phenotype gene set analysis of disease-based gene expression in mouse and human Physiol Genomics 2010 42A 2 162 167 10.1152/physiolgenomics.00008.2010 20682848 PMC2957794

[B54] Lu J Pazin MJ Ravid K Properties of ets-1 binding to chromatin and its effect on platelet factor 4 gene expression Mol Cell Biol 2004 24 1 428 441 10.1128/MCB.24.1.428-441.2004 14673175 PMC303331

[B55] Lee PP Fitzpatrick DR Beard C Jessup HK Lehar S Makar KW Perez-Melgosa M Sweetser MT Schlissel MS Nguyen S A critical role for dnmt1 and DNA methylation in T cell development, function, and survival Immunity 2001 15 5 763 774 10.1016/S1074-7613(01)00227-8 11728338

[B56] Strobeck MW DeCristofaro MF Banine F Weissman BE Sherman LS Knudsen ES The BRG-1 subunit of the SWI/SNF complex regulates CD44 expression J Biol Chem 2001 276 12 9273 9278 10.1074/jbc.M009747200 11108719

[B57] Wei L Vahedi G Sun HW Watford WT Takatori H Ramos HL Takahashi H Liang J Gutierrez-Cruz G Zang C Discrete roles of STAT4 and STAT6 transcription factors in tuning epigenetic modifications and transcription during T helper cell differentiation Immunity 2010 32 6 840 851 10.1016/j.immuni.2010.06.003 20620946 PMC2904651

[B58] Liao W Schones DE Oh J Cui Y Cui K Roh TY Zhao K Leonard WJ Priming for T helper type 2 differentiation by interleukin 2-mediated induction of interleukin 4 receptor alpha-chain expression Nat Immunol 2008 9 11 1288 1296 10.1038/ni.1656 18820682 PMC2762127

[B59] Ernst J Kellis M Discovery and characterization of chromatin states for systematic annotation of the human genome Nat Biotechnol 2010 28 8 817 825 10.1038/nbt.1662 20657582 PMC2919626

[B60] Tie F Banerjee R Stratton CA Prasad-Sinha J Stepanik V Zlobin A Diaz MO Scacheri PC Harte PJ CBP-mediated acetylation of histone H3 lysine 27 antagonizes Drosophila Polycomb silencing Development 2009 136 18 3131 3141 10.1242/dev.037127 19700617 PMC2730368

[B61] Jin Q Yu LR Wang L Zhang Z Kasper LH Lee JE Wang C Brindle PK Dent SY Ge K Distinct roles of GCN5/PCAF-mediated H3K9ac and CBP/p300-mediated H3K18/27 ac in nuclear receptor transactivation EMBO J 2011 30 2 249 262 10.1038/emboj.2010.318 21131905 PMC3025463

[B62] Creyghton MP Cheng AW Welstead GG Kooistra T Carey BW Steine EJ Hanna J Lodato MA Frampton GM Sharp PA Histone H3K27ac separates active from poised enhancers and predicts developmental state Proc Natl Acad Sci USA 2010 107 50 21931 21936 10.1073/pnas.1016071107 21106759 PMC3003124

[B63] Rada-Iglesias A Bajpai R Swigut T Brugmann SA Flynn RA Wysocka J A unique chromatin signature uncovers early developmental enhancers in humans Nature 2011 470 7333 279 283 10.1038/nature09692 21160473 PMC4445674

[B64] Zentner GE Tesar PJ Scacheri PC Epigenetic signatures distinguish multiple classes of enhancers with distinct cellular functions Genome research 2011 10.1101/gr.122382.111 PMC3149494 21632746

[B65] Wang Z Zang C Rosenfeld JA Schones DE Barski A Cuddapah S Cui K Roh TY Peng W Zhang MQ Combinatorial patterns of histone acetylations and methylations in the human genome Nat Genet 2008 40 7 897 903 10.1038/ng.154 18552846 PMC2769248

[B66] Ramos YF Hestand MS Verlaan M Krabbendam E Ariyurek Y van Galen M van Dam H van Ommen GJ den Dunnen JT Zantema A Genome-wide assessment of differential roles for p300 and CBP in transcription regulation Nucleic Acids Res 2010 38 16 5396 5408 10.1093/nar/gkq184 20435671 PMC2938195

[B67] Sahoo A Lee CG Jash A Son JS Kim G Kwon HK So JS Im SH Stat6 and c-Jun mediate Th2 cell-specific IL-24 gene expression J Immunol 2011 186 7 4098 4109 10.4049/jimmunol.1002620 21357535

[B68] Northrup DL Zhao K Application of ChIP-Seq and related techniques to the study of immune function Immunity 2011 34 6 830 842 10.1016/j.immuni.2011.06.002 21703538 PMC3137373

[B69] Memedula S Belmont AS Sequential Recruitment of HAT and SWI/SNF Components to Condensed Chromatin by VP16 Curr Biol 2003 13 3 241 246 10.1016/S0960-9822(03)00048-4 12573221

[B70] Hargreaves DC Crabtree GR ATP-dependent chromatin remodeling: genetics, genomics and mechanisms Cell research 2011 21 3 396 420 10.1038/cr.2011.32 21358755 PMC3110148

[B71] Ryme J Asp P Bohm S Cavellan E Farrants AK Variations in the composition of mammalian SWI/SNF chromatin remodelling complexes J Cell Biochem 2009 108 3 565 576 10.1002/jcb.22288 19650111

[B72] Biddie SC Hager GL Glucocorticoid receptor dynamics and gene regulation Stress 2009 12 3 193 205 10.1080/10253890802506409 19051126 PMC6319377

[B73] Clapier CR Cairns BR The biology of chromatin remodeling complexes Annu Rev Biochem 2009 78 273 304 10.1146/annurev.biochem.77.062706.153223 19355820

[B74] Ho L Crabtree GR Chromatin remodelling during development Nature 2010 463 7280 474 484 10.1038/nature08911 20110991 PMC3060774

[B75] Tarakhovsky A Tools and landscapes of epigenetics Nat Immunol 2010 11 7 565 568 10.1038/ni0710-565 20562839

[B76] Wysocka J Swigut T Xiao H Milne TA Kwon SY Landry J Kauer M Tackett AJ Chait BT Badenhorst P A PHD finger of NURF couples histone H3 lysine 4 trimethylation with chromatin remodelling Nature 2006 442 7098 86 90 16728976 10.1038/nature04815

[B77] Mendel I Shevach EM The IL-10-producing competence of Th2 cells generated in vitro is IL-4 dependent Eur J Immunol 2002 32 11 3216 3224 10.1002/1521-4141(200211)32:11<3216::AID-IMMU3216>3.0.CO;2-H 12555667

[B78] Zamisch M Tian L Grenningloh R Xiong Y Wildt KF Ehlers M Ho IC Bosselut R The transcription factor Ets1 is important for CD4 repression and Runx3 up-regulation during CD8 T cell differentiation in the thymus J Exp Med 2009 206 12 2685 2699 10.1084/jem.20092024 19917777 PMC2806616

[B79] Grenningloh R Miaw SC Moisan J Graves BJ Ho IC Role of Ets-1 phosphorylation in the effector function of Th cells Eur J Immunol 2008 38 6 1700 1705 10.1002/eji.200738112 18465773

[B80] Flowers S Nagl NG Jr Beck GR Jr Moran E Antagonistic roles for BRM and BRG1 SWI/SNF complexes in differentiation J Biol Chem 2009 284 15 10067 10075 10.1074/jbc.M808782200 19144648 PMC2665061

[B81] Hendriks RW Nawijn MC Engel JD van Doorninck H Grosveld F Karis A Expression of the transcription factor GATA-3 is required for the development of the earliest T cell progenitors and correlates with stages of cellular proliferation in the thymus Eur J Immunol 1999 29 6 1912 1918 10.1002/(SICI)1521-4141(199906)29:06<1912::AID-IMMU1912>3.0.CO;2-D 10382753

[B82] Pai SY Truitt ML Ting CN Leiden JM Glimcher LH Ho IC Critical roles for transcription factor GATA-3 in thymocyte development Immunity 2003 19 6 863 875 10.1016/S1074-7613(03)00328-5 14670303

[B83] Prlic M Bevan MJ Cutting Edge: {beta}-Catenin Is Dispensable for T Cell Effector Differentiation, Memory Formation, and Recall Responses J Immunol 2011 187 4 1542 1546 10.4049/jimmunol.1100907 21724993 PMC3150307

[B84] Weber BN Chi AW Chavez A Yashiro-Ohtani Y Yang Q Shestova O Bhandoola A A critical role for TCF-1 in T-lineage specification and differentiation Nature 2011 476 7358 63 68 10.1038/nature10279 21814277 PMC3156435

[B85] Chi TH Wan M Lee PP Akashi K Metzger D Chambon P Wilson CB Crabtree GR Sequential roles of Brg, the ATPase subunit of BAF chromatin remodeling complexes, in thymocyte development Immunity 2003 19 2 169 182 10.1016/S1074-7613(03)00199-7 12932351

[B86] Ji H Jiang H Ma W Johnson DS Myers RM Wong WH An integrated software system for analyzing ChIP-chip and ChIP-seq data Nat Biotechnol 2008 26 11 1293 1300 10.1038/nbt.1505 18978777 PMC2596672

[B87] Wei G Wei L Zhu J Zang C Hu-Li J Yao Z Cui K Kanno Y Roh TY Watford WT Global mapping of H3K4me3 and H3K27me3 reveals specificity and plasticity in lineage fate determination of differentiating CD4+ T cells Immunity 2009 30 1 155 167 10.1016/j.immuni.2008.12.009 19144320 PMC2722509

